# Ameliorative effect of bayberry leaves proanthocyanidins on high sugar diet induced *Drosophila melanogaster*

**DOI:** 10.3389/fphar.2022.1008580

**Published:** 2022-09-15

**Authors:** Mengting Wang, Haiguang Mao, Jianchu Chen, Lili Qi, Jinbo Wang

**Affiliations:** ^1^ School of Biological and Chemical Engineering, NingboTech University, Ningbo, China; ^2^ College of Biosystems Engineering and Food Science, Zhejiang University, Hangzhou, China

**Keywords:** bayberry leaves proanthocyanidins, hyperglycemia, drosophila, physiological markers, gene markers

## Abstract

Bayberry leaves proanthocyanidins (BLPs) were distributed in natural plant food, considered to have the potential for metabolic syndrome. In this study, we raised *Drosophila melanogaster* on high sugar diet (HSD) from the egg stage to induce hyperglycemia, and the ameliorative effect of BLPs was assessed based on this model. Phenotypical, biochemical, and molecular analyses related to diabetes mellitus pathogenesis were measured. Flies exposed to BLPs were found to suppress the HSD-induced high glucose and high triglycerides levels. Moreover, BLPs showed an inhibitory effect on carbohydrate digestive enzymes (α-amylase and α-glucosidase) activity and mRNA expression, exhibiting the potential for carbohydrate digestion retardation. Transcriptional levels of key genes associated with glycolipid metabolism were further evaluated, including *dilp*, *InR,* and downstream *dAKT-dFOXO-PEPCK*, together with *E78*, *SREBP*, *FAS*, and *LSD* genes, were all downregulated after BLPs-exposure, suggesting the ameliorative effect of BLPs on dysbiosis associated with the insulin signaling pathway. This study provided a new functional compound, which is beneficial to further antidiabetic therapy studies.

## Introduction

Diabetes mellitus is a chronic metabolic syndrome as well as an incapacitating disease. It has been reported that millions of adults suffer from diabetes mellitus, and 90% of those are type 2 diabetes mellitus (T2DM) (Lingvay et al., 2022). T2DM, the target cells of insulin fail to respond to the hormone, is often accompanied by a host of pathologies, including obesity, fatty liver, cardiovascular disease, and nephropathy. The imbalance of glucose homeostasis with insulin resistance is one of the hallmarks of T2DM (Roden and Shulman, 2019).

Proanthocyanidins were widely found in many plants and their derived foods, which had a beneficial effect on T2DM, largely free from side effects (Zeng, et al., 2020). Our previous studies found that proanthocyanidins from bayberry (*Myrica rubra* Sieb. et Zucc.) leaves (BLPs) belonged to prodelphinidins with a potent EGCG unit and a mean degree of polymerization (mDP) of about 6.5, which were different with the structural characteristics of those common procyanidins (Tao et al., 2020). Furthermore, *in-vitro* experiments have been conducted to evaluate the hypoglycemic potential of BLPs, which were reported to have an advantage in inhibiting α-glucosidase activity and lowering glucose consumption (Wang et al., 2019) (Zhang et al., 2017). BLPs can also effectively reduce the risk related to metabolic disorders in high-fat diet induced obese mice (Zhang et al., 2022). However, the antidiabetic activity of BLPs and their underlying molecular mechanism is necessary to study.

*D. melanogaster* was increasingly used as a valuable invertebrate model for understanding T2DM (Álvarez-Rendón et al., 2018). *Drosophila* has organs of the heart, brain, kidney (nephrocytes, Malpighian tubules), liver, fat tissue, gastrointestinal tract, and blood (hemolymph), which contribute to the conserved effect and mechanism in energy metabolism and glucose homeostasis analogous to humans (Pendse et al., 2013). The insulin signaling pathways are closely related to maintaining the glucose homeostasis of *Drosophila*, which is highly conserved during evolution (Rulifson et al., 2002). Insulin-like peptides in *Drosophila* (dilps) are equivalent to the vertebrate insulin-like growth factor, affecting the insulin signaling pathway and regulating growth and glucose homeostasis. And insulin signaling in flies follows the same canonical pathway as mammals: insulin-receptor (InR) activation stimulates the downstream AKT-TOR-FOXO signaling (Semaniuk et al., 2021). In addition, *Drosophila* offers a simpler animal system that allows the molecular mechanisms of gene function to be readily manipulated throughout the lifecycle in comparison to vertebrate models. Therefore, the low cost and rapid generation time of *Drosophila* make an efficient contribution to the *in-vivo* investigation.

Recently, high-calorie diet feeding was usually used to induce metabolic disorders (such as insulin resistance or obesity) in *Drosophila*, which was close to the actual situation. Musselman et al. (2011) found that *Drosophila* larvae reared on excess sugar (maltose) diet, elicited hyperglycemic and insulin-resistant phenotypes and upregulated expression of genes involved in lipogenesis, gluconeogenesis, and β-oxidation. Based on such a high sugar diet (HSD) or high fat diet (HFD) challenged *Drosophila* model, the role of some dietary food in anti-diabetic and anti-obesity therapies were evaluated, such as tea polyphenols (Kayashima et al., 2015), *Syzygium cumini* and *Bauhinia forficate* (Ecker et al., 2017), *Flos Chrysanthemi Indici* extract (Bai et al., 2018), vitamin B6 (Mascolo et al., 2022), and so on.

In this study, HSD feeding was applied to induce hyperglycemic *Drosophila* flies. HSD-fed flies were subsequently exposed to a BLPs-supplemented diet at two stages, to investigate whether BLPs had an ameliorative effect on T2DM-like phenotypes. We hope to provide a new therapy of BLPs for a dietary challenged model with disruption of glucose homeostasis.

## 2 Materials and methods

### Fly stock and culture

The *D. melanogaster* (w^1118^ strain) was obtained from the Core Facility of *Drosophila* Resource and Technology, Shanghai Institute of Biochemistry and Cell Biology, CAS. The flies were cultured on normal diet (ND) containing 10.5% (w/v) corn meal, 7.5% (w/v) sucrose, 4% (w/v) yeast, 0.75% (w/v) agar, and 1% (v/v) propionic acid and maintained in an incubator (25 ± 1°C; relative humidity of 60%; 12-h dark/light cycle). The high-sugar diet (HSD) was prepared made up of 10.5% (w/v) corn meal, 30% (w/v) sucrose, 4% (w/v) yeast, 0.75% (w/v) agar, and 1% (v/v) in distilled water.

### Bayberry leaves proanthocyanidins preparation

BLPs were extracted from dried bayberry leaves powder (Cixi, Zhejiang Province, China) and purified by HPD-500 column and Sephadex LH-20 column according to our laboratory methods (Zhang et al., 2017), the structural information of which were shown in Supplementary Materials (Supplementary Figure S1; Supplementary Table S1).

### Experiment design

**FIGURE 1:**
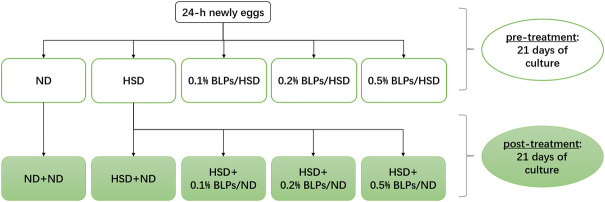
Experiment design of BLPs supplementation in two treatments.

In this study, we exposed HSD-fed flies to BLPs in two ways, including pre-treatment and post-treatment (Figure 1 **)**. At the pre-treatment stage, 24 h-newly eggs were treated with the following diets for 21 days passing through embryonic and larval stages to adult flies: ND, HSD, HSD supplemented with 1 mg/ml BLPs (0.1% BLPs/HSD), HSD supplemented with 2 mg/ml BLPs (0.2% BLPs/HSD), HSD supplemented with 5 mg/ml BLPs (0.5% BLPs/HSD), respectively. Thereafter, at the post-treatment stage, ND flies and HSD flies were treated with the following diets for another 21 days: ND flies reared on ND (ND+ND), HSD flies reared on ND (HSD+ND), HSD flies reared on ND supplemented with 1 mg/ml BLPs (HSD+0.1% BLPs/ND), HSD flies reared on ND supplemented with 2 mg/ml BLPs (HSD+0.2% BLPs/ND), HSD flies reared on ND supplemented with 5 mg/ml BLPs (HSD+0.5% BLPs/ND), respectively.

### Developmental rate and pupation morphology

Growth behaviors of HSD-fed *Drosophila* in the periods of egg-adult were recorded using photographs every day, and the first time observed larvae, pre-pupa, pupa, and flies were recorded as well. Meanwhile, the pupas climbed up the walls were collected using a brush and then mounted onto the slide using glycerin for microscope photography (Jun et al., 2016).

### Body weight of flies

At the end of each experiment, flies were transferred into a pre-weighed EP tube under brief anesthesia. The total body weight of 10 flies per group was assessed using the microbalance.

### Glucose and triglyceride level

Glucose, triglyceride (TAG) and protein levels were estimated using Glucose Assay Kit (GOPOD format) (Megazyme, Ireland), Triglyceride Assay Kit (GPO-PAP format) (Nanjing Jiancheng Bioengineering Institute, China), and BCA protein Assay Kit (Beyotime Institute of Biotechnology, China) following manufacturer’s protocols. Sample preparation was followed by the previous report with some modifications (Westfall et al., 2018). For homogenate extraction, five flies were liquid nitrogen frozen and homogenized in 300 μl PBS. After centrifuging at 12,000 rpm for 10 min, 2 μl of supernatant was obtained for TAG content assay and 20 μl of supernatant was used for protein content determination. Following, the homogenate was heat-treated for 20 min at 70°C to remove any complexes. After centrifuging, the body glucose content was obtained and analyzed. Quantities of glucose and triglyceride were both standardized against the corresponding protein content to account for variations in fly mass.

### Activity assay of α-amylase and α-glucosidase

The α-amylase and α-glucosidase activity assays were carried out according to the previous report (Oboh et al., 2019). Flies were liquid nitrogen frozen, homogenized in PBS, and centrifuged (12,000 rpm, 10 min) to obtain supernatant. The supernatant was pre-incubated with the substrate (starch paste or pNPG), and the enzymatic reaction began immediately once the enzyme solution was added. After incubation at 37°C for 5 min, the absorbance was measured. The digestive enzyme activity was standardized against the protein content.

### Real-time quantitative PCR assay

Total RNA was extracted from frozen flies with Trizol reagent (GenStar Biosolutions Co., Ltd, China) following the manufacturer’s protocol (DeAngelis and Johnson, 2019). The purity and content of RNA were measured by NanoDrop spectrophotometer (Thermo Fisher Scientific Inc., United States). Reverse transcription was then carried out from isolated RNA using PrimeScript RT reagent Kit with gDNA Eraser Kit (Takara Bio Inc., China). Synthesized cDNA was used as the template for expression profiling, together with specific primers and PowerUp SYBR Green Master Mix (Applied Biosystems, United States) through Quant Studio 3 Real-Time PCR System (Applied Biosystems, United States). The relative transcription level of candidate genes was calculated using the 2^−ΔΔCt^ equation and rp49 (internal control). Specific primers in this study were listed in Table 1.

**TABLE 1:** Primer of specific genes used for qPCR.

Gene name	Sequence 5′–3′	Annealing temp (°C)
*Amy*	Forward: TCC​TTC​TCC​TTC​ACG​GAC​AC	54
	Reverse: TGC​AGG​AGT​TGT​CGC​TAT​TG	
*Mal-5b*	Forward: CAA​CAC​CAA​TCC​CAG​CAT​CT	54
	Reverse: CTG​CTC​CAC​ATT​CAC​CTC​CT	
*dilp 2*	Forward: AGC​AAG​CCT​TTG​TCC​TTC​ATC​TC	58
	Reverse: ACA​CCA​TAC​TCA​GCA​CCT​CGT​TG	
*dilp 3*	Forward: TGT​GTG​TAT​GGC​TTC​AAC​GCA​ATG	60
	Reverse: CAC​TCA​ACA​GTC​TTT​CCA​GCA​GGG	
*InR*	Forward: AAC​AGT​GGC​GGA​TTC​GGT​T	56
	Reverse: TAC​TCG​GAG​CAT​TGG​AGG​CAT	
*MAPK*	Forward: CAA​TCG​CCC​ACC​TAA​ACA​AAA	56
	Reverse: GCC​CAA​CTT​CTC​CAA​TGA​CC	
*FAS*	Forward: CAA​CAA​GCC​GAA​CCC​AGA​TCT​T	56
	Reverse: CAA​AGG​AGT​TCA​GGC​CGA​TGA​T	
*PEPCK*	Forward: CGC​CCA​GCG​ACA​TGG​ATG​CT	58
	Reverse: GTA​CAT​GGT​GCG​ACC​CTT​CA	
*dTOR*	Forward: GGC​CGT​CCA​GGT​TCA​AAA​AC	55
	Reverse: AAT​CCG​GCG​ATA​GTT​CCG​TC	
*dAKT*	Forward: GAG​TCG​TGT​GCT​CAA​GTC​CA	55
	Reverse: TGC​ATC​ACA​AAA​CAC​AGG​CG	
*dFOXO*	Forward: TCG​CCG​AAC​TCA​GTA​ACC​AC	55
	Reverse: TCC​TAT​CAA​AGT​AGA​GGC​GCA	
*SREBP*	Forward: GGC​AGT​TTG​TCG​CCT​GAT​G	56
	Reverse: CAG​ACT​CCT​GTC​CAA​GAG​CTG​TT	
*LSD*	Forward: ACT​TGT​AGT​GCC​AGT​TCC​CG	55
	Reverse: ACC​AGA​CTG​CTC​CAC​ATT​CG	
*E78*	Forward: CAG​TGT​CTC​TCG​TTG​CTC​A	53
	Reverse: AAC​CGA​TTG​CTT​CGC​TCT​CT	
*rp49*	Forward: AGA​TCG​TGA​AGA​AGC​GCA​CCA​AG	58
	Reverse: CAC​CAG​GAA​CTT​CTT​GAA​TCC​GG	

### Statistical analysis

Data were expressed as the mean value ±standard deviation. Analysis of variance (ANOVA) with Duncan’s difference analysis was applied to all data using Version 20.0 SPSS Statistics (SPSS Inc., Chicago, IL).

## Results and discussion

### High sugar diet induced T2DM-like phenotype of drosophila

In this study, *Drosophila* offspring were reared on a high-sugar diet (HSD) starting from eggs to simulate T2DM. To explore the appropriate concentration of HSD (20%–40% sucrose), growth behavior including developmental rate and pupation morphology of *Drosophila* were observed.

**FIGURE 2:**
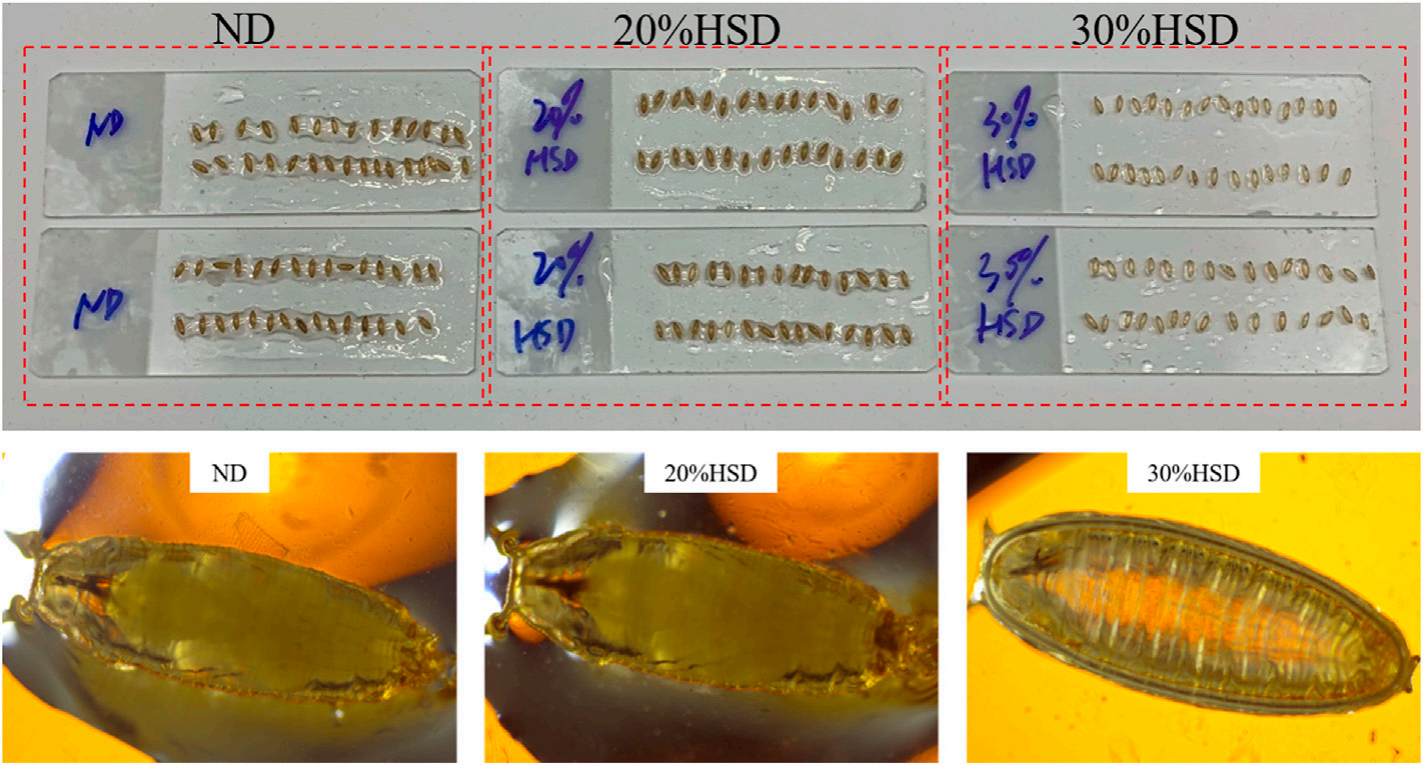
Photographs by camera and microscope (20×) of pupa after HSD feeding.

Ordinarily, in the fly life cycle, eggs laid by adult flies hatch into larvae and grow into first-instar and second-instar larvae in 5–6 days. At the stage of the third instar, larvae begin to leave the food and “wander” to the wall. After about 11–12 days, third-instar larvae metamorphose into pre-pupa and pupa and then adult flies (Abrat et al., 2018). In the present study, we observed that the growth process of normal diet (ND) fed flies was broadly consistent with expectations, but that of 20% or 30% HSD fed flies had a delay of 2–3 days whether in the larval stage or pupa stage. Meanwhile, a sugar tolerance with growth retardation happened in 40% HSD intake, which was observed to stop growing at the stage of third-instar larvae and even cannot reach the pupal state (Supplementary Figure S2). Furthermore, pupa treated with 30% HSD were observed to show a more “transparent-like” appearance (Figure 2), which tended to be in the pre-pupa stage, indicating that 30% of sucrose induced a more severe growth-deficiency phenotype during the growth and development period of *Drosophila*.

Besides developmental delay, 30% HSD fed-flies exhibited an evident increase in body glucose and triglyceride level (Supplementary Figure S3), as well as carbohydrate digestive enzymes activities (Supplementary Figure S4). Subsequently, the mRNA levels of genes involved in glucose and lipid metabolism significantly altered in flies with HSD consumption (Supplementary Figure S5). Our results revealed that 30%HSD intake led to the pathophysiological and transcriptional changes in *Drosophila*, which are consistent with the T2DM phenotype as previously reported (Meshrif et al., 2022) (Eickelberg et al., 2022). In the present study, HSD participated the growth and development stages of *Drosophila*, therefore affecting the organ development and insulin action, which in turn influenced the glucose homeostasis and insulin sensitivity of flies (Cassim et al., 2018).

### Physiological markers associated with hyperglycemia in high sugar diet-fed drosophila exposed to bayberry leaves proanthocyanidins

**FIGURE 3:**
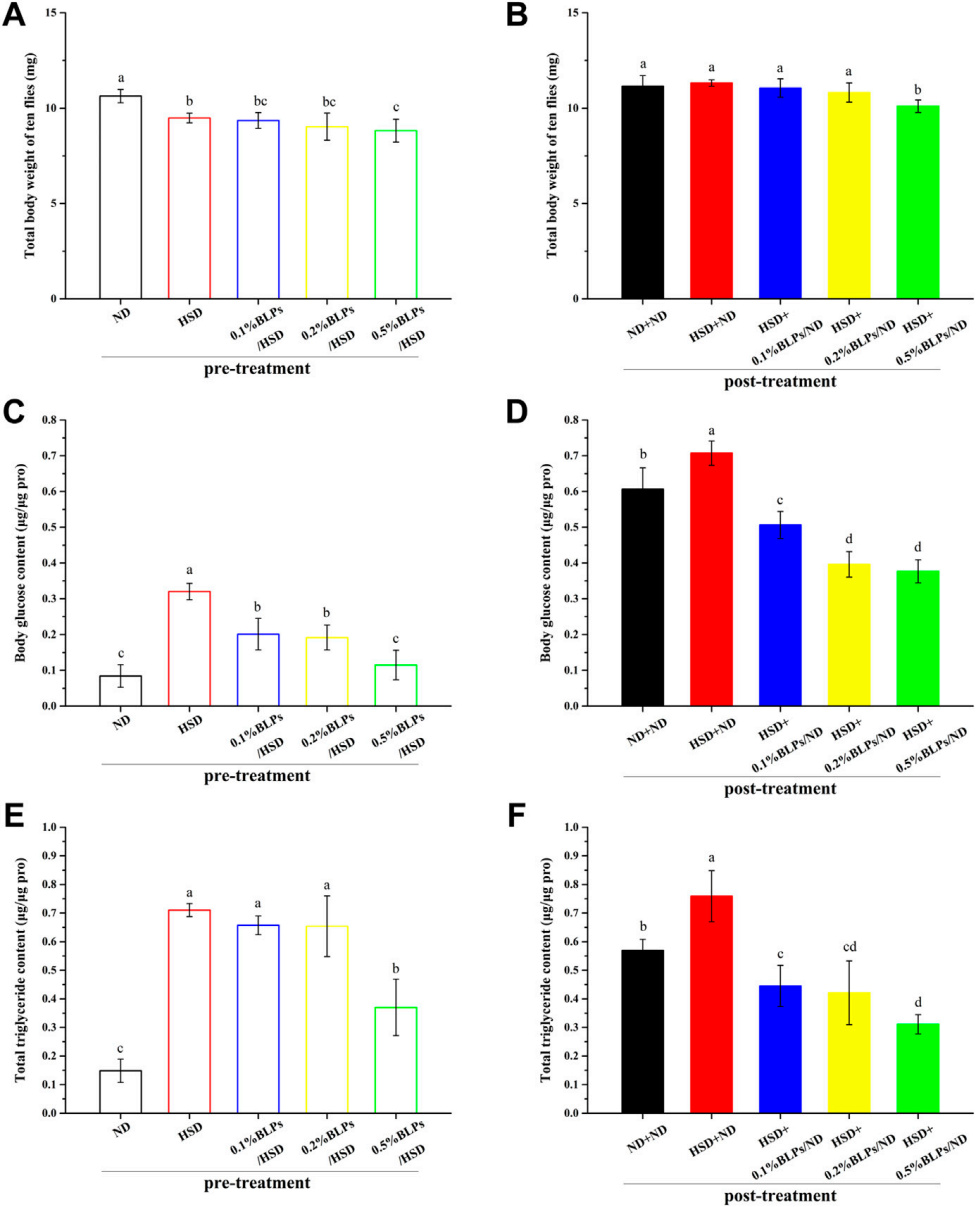
Body weight (ten flies) **(A,B)**, body glucose content **(C,D)**, and total triglyceride content **(E,F)** of those flies fed on a diet containing BLPs at different concentrations in pre-treatment or post-treatment, respectively. Values with different letters **(A–D)** represent significant differences among the groups (*p* < 0.05).

To examine the ameliorative effect of BLPs on physiological markers associated with hyperglycemia in *Drosophila*, the body weight, and the levels of glucose and triglyceride were determined (Figure 3).

As can be observed in Figure 3A, flies exposed to BLPs supplemented with HSD appeared a slight weight loss compared to HSD-fed counterparts in pre-treatment. In post-treatment (Figure 3B), a high dosage of 0.5% BLPs consumption led to a 10.73% weight loss in HSD flies. A similar result was observed in green tea polyphenols (GTP), which were reported to reduce the size and weight of *Drosophila* at the 2.5–10 mg/ml doses of GTP, was the result of reduced cell size or cell numbers (Lopez et al., 2016). Previous findings illustrated that body weight and visceral index in HFD mice decreased after a 4-week intervention of BLPs (Zhou et al., 2017).

Moreover, we observed that the elevated total body glucose and triglyceride levels in HSD-fed flies were diminished with the administration of BLPs. In detail, those flies treated with BLPs supplemented with HSD had a great reduction in body glucose level by 64.08% (Figure 3C) and in triglyceride level by 47.92% occurring at 0.5% BLPs (Figure 3E). In post-treatment, HSD induction was no longer continued, and the hyperglycemia and accumulation of lipid have improved but remained. HSD+ND group had 1.17-fold higher levels of body glucose and 1.33-fold higher levels of total triglyceride compared to the ND+ND group. After BLPs intervention, flies exhibited hypoglycemic and lipid-lowering effects, with a reduction in body glucose level by 46.78% (Figure 3D) and in triglyceride level by 59.01% occurring at 0.5% BLPs (Figure 3F). The HSD-induced glucose and triglyceride levels were reported to significantly lowered in w^1118^ flies exposed to HSD supplemented with *Solanum anguivi* Lam. fruit (Nakitto et al., 2021) and Avens root extract (Günther et al., 2021), respectively.

Our findings indicated that the administrated BLPs whether in pre-treatment or post-treatment had an alleviative effect on HSD-induced accumulation of glucose and triglyceride, considered an anti-diabetic property (Alfa and Kim, 2016). It was consistent with the previous reports in rodent models that BLPs supplementation significantly reduced the blood glucose and AUC of OGTT (Zhang et al., 2022), as well as serum total cholesterol (TG) and triglyceride (TC) content in high-fat-fed rats (Zhou et al., 2017). To further investigate the hypoglycemic mechanism of BLPs, we focused on the role of BLPs in starch digestion and the glucose metabolism process, contributing to glucose homeostasis in HSD-fed flies.

### Activity and mRNA expression of digestive enzymes in high sugar diet-induced drosophila exposed to bayberry leaves proanthocyanidins

α-Amylase and α-glucosidase are considered the major carbohydrate hydrolyzing enzymes in *Drosophila*, same as human beings. The starch and other polysaccharides in the diet were hydrolyzed to disaccharides by pancreatic α-amylase, followed by being digested to glucose by α-glucosidase in the small intestine. Therefore, the activity of these enzymes is relevant to the glucose release from carbohydrates in the diet (Sun and Miao, 2020).

**FIGURE 4:**
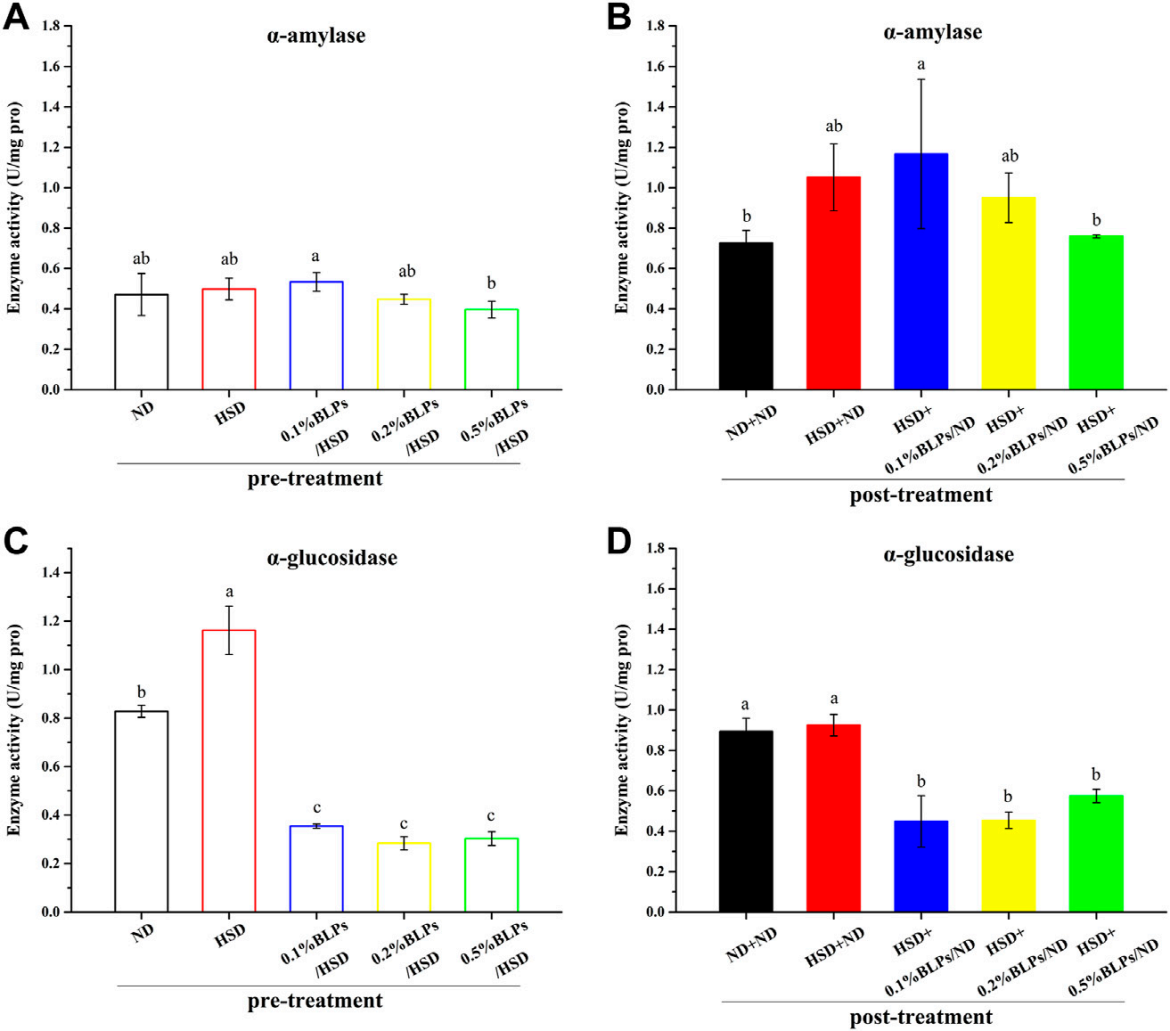
α-Amylase activity **(A,B)** and α-glucosidase activity **(C,D)** of those flies fed on a diet containing BLPs at different concentrations in pre-treatment or post-treatment, respectively. Values with different letters **(A–D)** represent significant differences among the groups (*p* < 0.05).

The activity of two typical carbohydrate digestive enzymes (α-amylase and α-glucosidase) in flies was shown in Figure 4. Our findings showed that HSD feeding significantly simulated α-glucosidase activity. Similar results were observed in the report of Oyeniran et al. (2020), which indicated that flies fed with a diet supplemented with 30% sucrose obviously increased the digestive enzyme activity. This report was inclined to the view that the changes in enzyme activity reflected a change in enzyme quantity rather than a change in catalytic efficiency in the presence of high sugar. In this study, HSD caused a relative increase in α-glucosidase activity in response to the increase in sucrose (as substrate) amount. Moreover, carbohydrate digestive enzyme activities were closely associated with glucose homeostasis in *Drosophila* flies (Bezzar-Bendjazia et al., 2017). The higher activities of digestive enzymes after HSD-feeding could be one explanation for the dysglycemia as above mentioned.

With the exposure of BLPs, it seemed that BLPs inhibited α-amylase and α-glucosidase activity to some extent, indicating the hypoglycemic potential. In detail, 0.5% of BLPs inhibited α-amylase activity by 20.47% but were not significant in pre-treatment, while greatly inhibited α-amylase activity by 27.79% in post-treatment. Effective inhibition of α-glucosidase activity has been achieved at the low dosage of 0.1% BLPs, around a 73.95% reduction in pre-treatment and a 51.55% reduction in post-treatment. Comparatively, BLPs had a more dramatic inhibition effect on α-glucosidase activity than α-amylase activity. This result was following our previous *in-vitro* studies, which suggested that BLPs had the inhibitory activity to α-glucosidase of 517.01 mM acarbose equivalents/g extract (Wang et al., 2019) and the inhibitory activity to α-amylase of 2.92 mM acarbose equivalents/g extract (Wang et al., 2020), respectively. The inhibitory effect of BLPs on the activity of these two enzymes was mainly attributed to affecting the conformational structure and micro-environment of the enzymes through BLPs-enzyme interaction.

**FIGURE 5:**
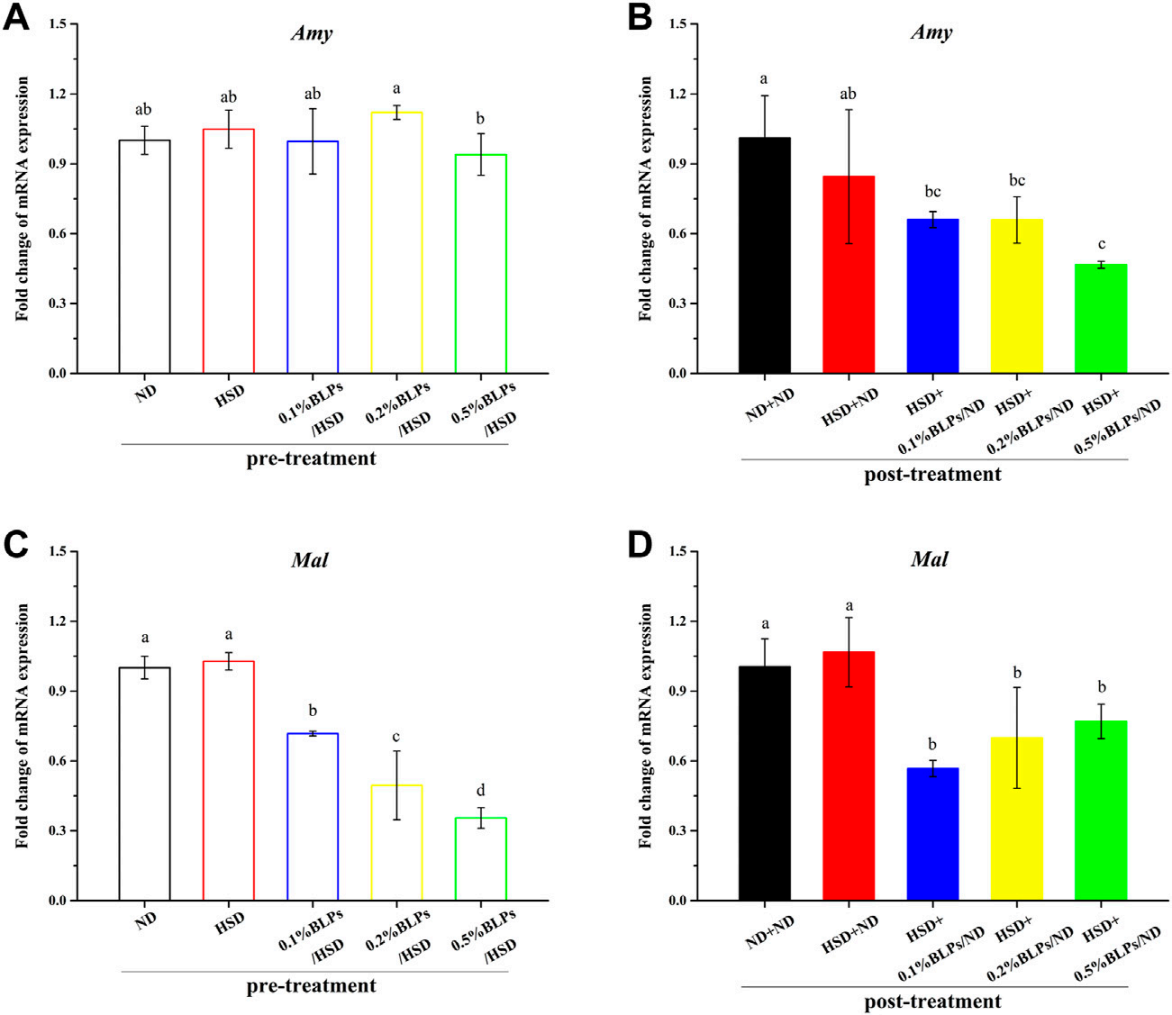
mRNA expression of Amy gene **(A,B)** and Mal-5b gene **(C,D)** of those flies fed on media containing BLPs at different concentrations in pre-treatment and post-treatment, respectively. Values with different letters **(A–D)** represent significant differences among the groups (*p* < 0.05).

Further, previous reports suggested that the digestive enzyme activity is involved in the transcription level of multi-duplicated genes. *Drosophila* genomes involved *Amy* (Amy-alleles and Amy-genotypes) and ten duplicated *Mal* genes, and these genes were regulated and associated with carbohydrate changes in the food substrate (Inomata et al., 2019), (Lai et al., 2014). In this study, the expression levels of the main digestive enzymes genes (Amy and Mal-5b) were investigated (Figure 5). mRNA expressions of *Amy* and *Mal-5b* in the BLPs-exposed flies were both downregulated, which led to the changes in enzyme activity. The retardation of BLPs on carbohydrate digestion is one of the main ways to achieve hypoglycemic action, especially through inhibiting the α-glucosidase activity.

### Gene markers of glucose metabolism in high sugar diet-induced drosophila exposed to bayberry leaves proanthocyanidins

**FIGURE 6:**
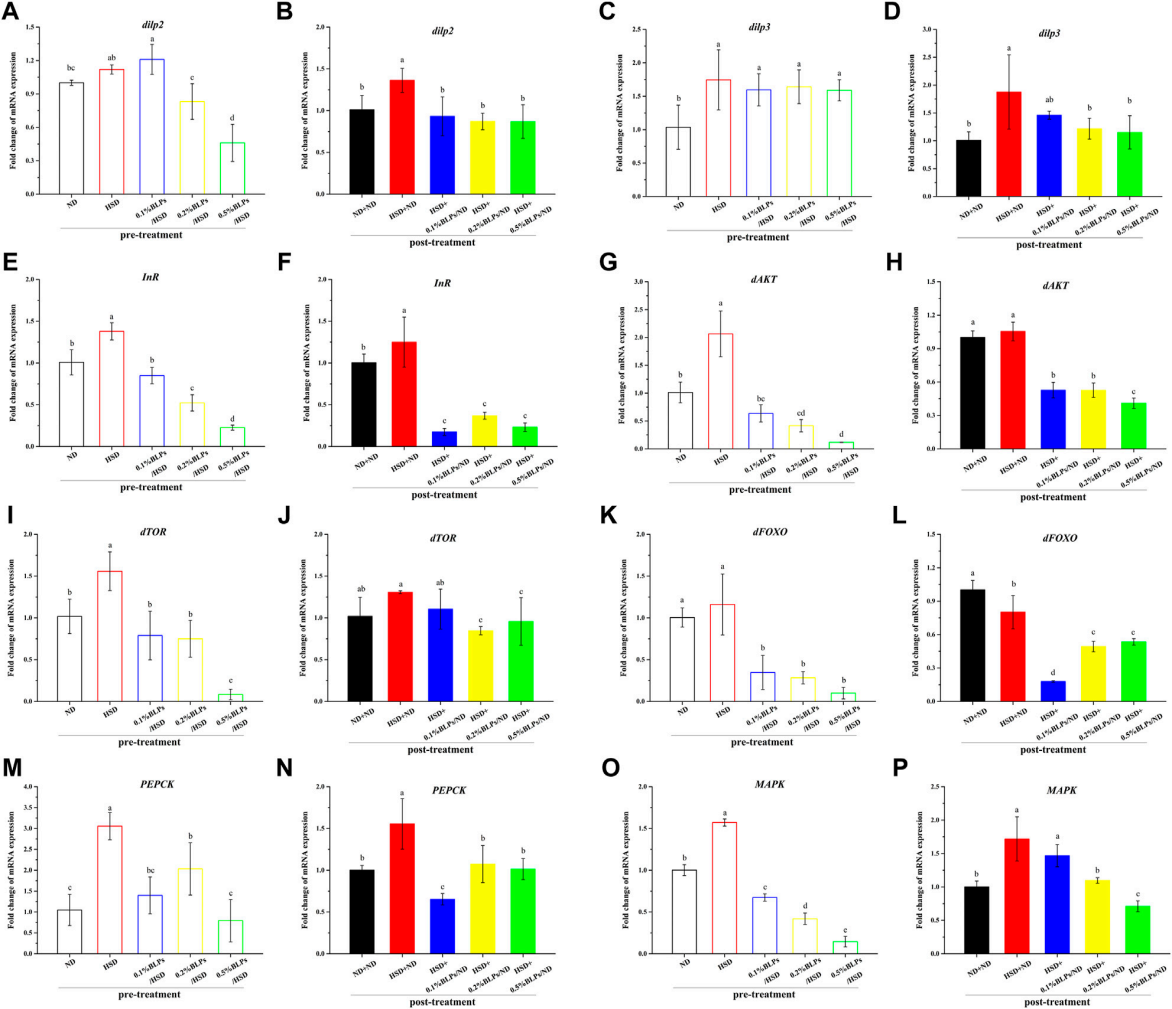
mRNA expression of main genes associated with glucose metabolism of those flies fed on media containing BLPs at different concentrations. Values with different letters **(A–P)** represent significant differences among the groups (*p* < 0.05).

Regulation of the insulin signaling pathway was closely relevant to glucose metabolism. To further determine whether BLPs made a difference in glucose metabolism in *Drosophila*, the transcriptional level of some key genes regulating the insulin signaling pathway was accessed (Figure 6).

As can be observed in Figures 6A–D, mRNA expression of energy metabolism regulators, insulin like-peptide 2 and 3 (*dilp2* and *dilp3*), were elevated in HSD-fed flies compared to ND-fed flies, while decreased after BLPs exposure. At the dosage of 5 mg/ml, BLPs greatly downregulated *dilp2* and *dilp3* levels to 0.41-fold and 0.91-fold that of the HSD group in pre-treatment, and to 0.64-fold and 0.61-fold that of the HSD+ND group in post-treatment. Moreover, insulin receptor (*InR*) transcript level also showed an increase in HSD flies, which had a remarkable down-regulation in BLPs-exposed flies (Figures 6E,F). It exhibited a dose-dependent decrease in pre-treatment, with a maximum drop ratio of 83.60%. However, there was no significant difference between the multiple dosages of BLPs, accompanied by a 70.54%–86.04% decrease.

Correspondingly, transcript levels of insulin-signaling regulators-protein kinase B (*dAKT*) and protein synthesis regulators-target of rapamycin (*dTOR*) were significantly upregulated, with a slight increase in Forkhead Box-O (*dFOXO*) in response to HSD feeding in pre-treatment, whereas dose-dependent downregulated in BLPs-exposed flies. And with a cessation of HSD induction in post-treatment, elevated mRNA expressions of *dAKT*, *dTOR*, and *dFOXO* in HSD flies have been impaired, which were further decreased when flies were reared on a BLPs-supplemented diet (Figure 6G–L). What’s more, phosphoenolpyruvate carboxykinase (*PEPCK*) is regulated by dFOXO activation. Elevated *PEPCK* expression in HFD flies significantly downregulated in the presence of BLPs (Figure 6M–N), reflecting inhibition of gluconeogenesis. Mitogen-activated protein kinase (MAPK), a key regulator of energy metabolism, was also reduced in mRNA expression by BLPs.

Insulin resistance is regarded as an over-production of insulin due to the cells’ reduced sensitivity to insulin (Westfall et al., 2018), which is related to the imbalance in insulin signaling pathway regulation. Generally, the insulin signaling pathway in *Drosophila* involves the following links. Insulin-like peptides (dilps) are secreted by the insulin-producing cells (IPCs), which subsequently bind to the single insulin receptor (InR) to activate the insulin/insulin-like growth factor signaling pathway (IIS) (Tatar et al., 2001). As a result, InR is activated, and thus leads to the stimulation of dAKT along with the activity regulation of the downstream dTOR pathway, and the subsequent sequestration of transcription factor dFOXO activity (Loreto et al., 2021). Considering that the mRNA expression of *dilp2*, *dilp3*, *InR*, and downstream *dAKT-dTOR* were all upregulated in HSD flies, it conformed to the insulin resistance-like (Nayak and Mishra, 2021). Our results illustrated that HSD feeding increased the signs of glucose metabolic stress in statistics and successfully induced insulin resistance based on mRNA expression whether in pre-treatment or post-treatment. And decreased *dilp*, *InR*, *dAKT*, and *dTOR* mRNA expression in BLPs-exposed flies were the consequence of reduced insulin signaling, suggesting the ameliorative effect of BLPs on HSD-induced insulin-resistance. Numerous studies have demonstrated the hypoglycemic action of some proanthocyanidins, such as grape seed procyanidins (Montagut et al., 2010), apple procyanidins (Ogura et al., 2016), proanthocyanidins from *I. lacteals* (Tie et al., 2020). They were reported to alleviate insulin resistance in type 2 diabetes mellitus *via* modulating second messenger signaling pathways, including PI3K-AKT, MAPK, and JNK signaling. What’s more, BLPs downregulated *dFOXO-PEPCK* expression, resulting in gluconeogenesis inhibition, which subsequently contributed to a reduction of glucose output (Gu et al., 2018).

### Gene markers of lipid metabolism in high sugar diet-induced drosophila exposed to bayberry leaves proanthocyanidins

**FIGURE 7:**
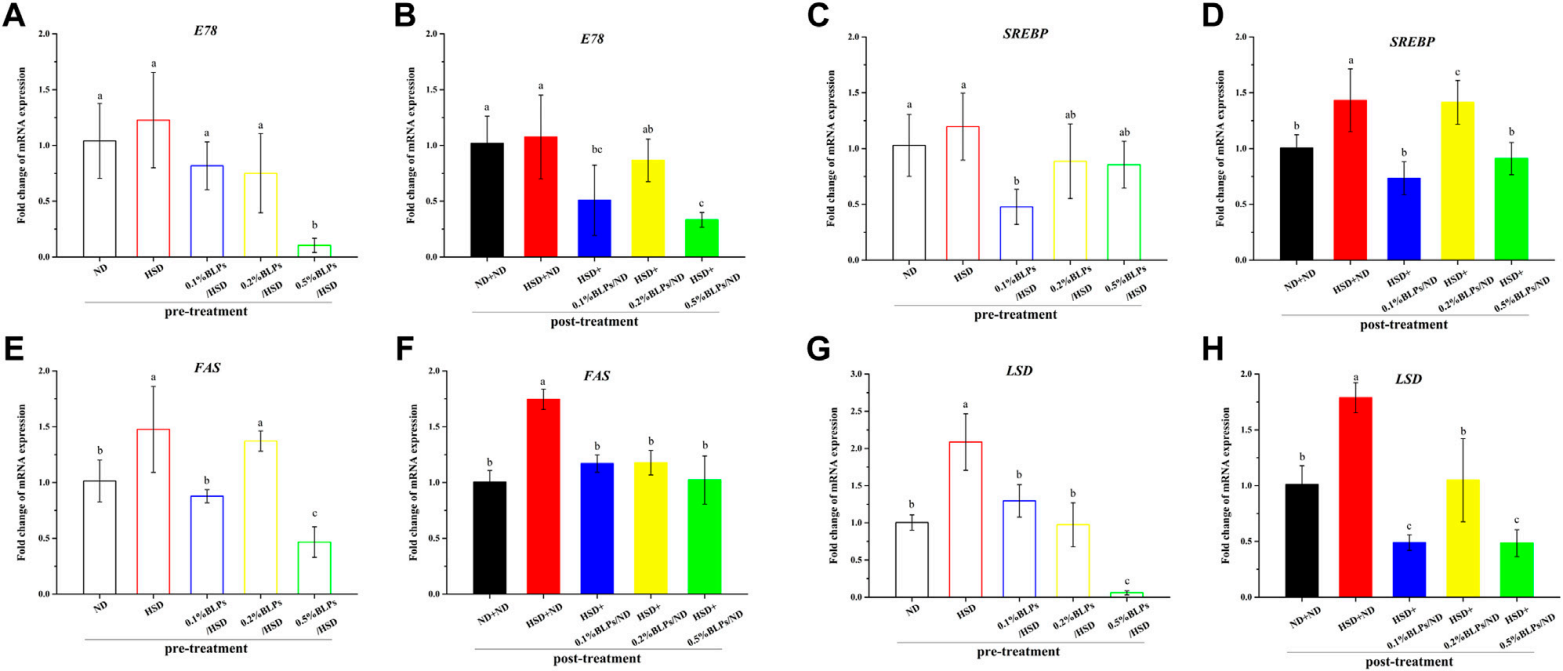
mRNA expression of main genes associated with lipid metabolism of those flies fed on media containing BLPs at different concentrations. Values with different letters **(A–H)** represent significant differences among the groups (*p* < 0.05).

Dysbiosis associated with the insulin signaling pathway would lead to insulin resistance accompanied by obesity in general (Reynés et al., 2017), so the transcriptional level of some key genes related to obesity was also investigated (Figure 7). E78 as the downstream transcriptional target of peroxisome proliferator-activated receptor-γ (PPARγ), mRNA expression of which was down-regulated with BLPs-exposure (Figure 7A–B). In parallel, as the key lipogenesis regulator, mRNA expression of sterol-regulatory element binding protein (*SREBP*) was slightly upregulated in HSD-fed flies compared to ND-fed flies in pre-treatment. It was aggravated in post-treatment but improved with BLPs treatment (Figure 7C–D). In terms of fatty acid synthase (*FAS*) and lipid storage droplet 2 (*LSD*), their transcript level was elevated with HSD feeding, whereas impaired in flies exposed to BLPs.

PPARγ is known as an important regulator of cholesterol, lipid and glucose metabolism (Han et al., 2019). With the activation of PPARγ, the mRNA expression of the downstream gene (*E78*) and adipogenic and lipogenic gene (such as *SREBP*) would be in turn stimulated, therefore leading to lipid storage, fatty acid oxidation, triglyceride synthesis as well as insulin sensitivity (Evans et al., 2013). SREBP is also the master transcriptional regulator of lipogenic enzymes (e.g. FAS), committed steps of cholesterol or fatty acid synthesis (Bertolio et al., 2019). And LSD plays a key role in lipid storage control (Kühnlein, 2012). Our results showed that HSD treatment upregulated the mRNA expression of *SREBP*, *FAS*, and *LSD*, which was the markers of aggravated obesity, including lipid storage, triglyceride synthesis and insulin resistance. As expected, BLPs markedly attenuated these dyslipidemia symptoms induced by HSD consumption. BLPs reducing oleic acid-induced lipid accumulation were previously observed in HepG2 cells, by modulating the expression of proteins related to TAG biosynthesis and sterols (Zhang et al., 2017).

## Conclusion

**FIGURE 8:**
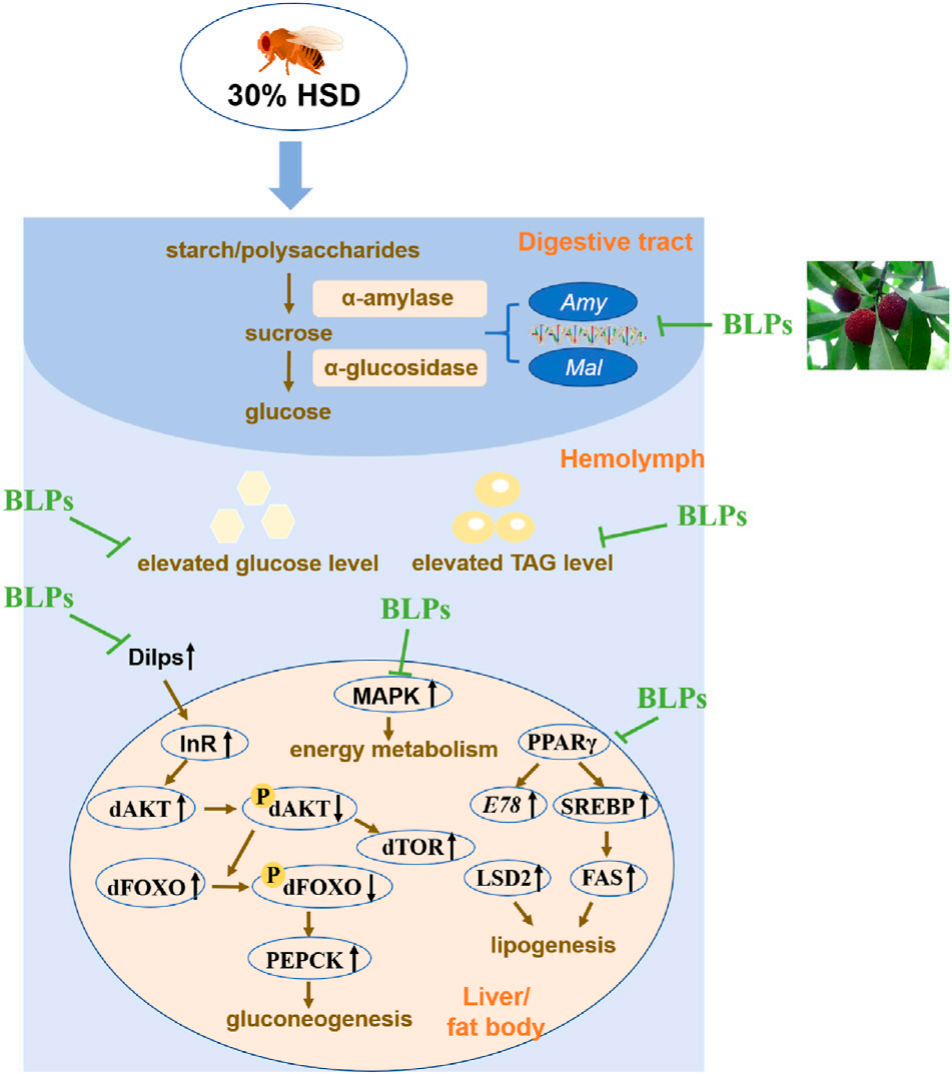
Schematic diagram of BLPs regulating T2DM-like phenotypes in HSD-fed flies. In brief, BLPs can effectively retard carbohyrate digestion and modulate the key proteins in glucolipid metabolism pathway, contributing to decrease the elevated glucose and TAG levels in HSD-fed flies. Dilps, *Drosophila* insulin-like peptides; InR, insulin-receptor; dAKT, protein kinase B in *Drosophila*; dTOR, target of rapamycin in *Drosophila*; dFOXO, forkhead box-O in *Drosophila*; PEPCK, phosphoenolpyruvate carboxykinase; MAPK, mitogen-activated protein kinase; PPARγ, peroxisome proliferator-activated receptor-γ; SREBP, sterol-regulatory element binding protein; FAS, fatty acid synthase; LSD2, lipid storage droplet 2.

In this study, we sought to utilize *Drosophila* fed a 30% high sugar diet to induce T2DM-like flies. Our results demonstrated that 30% HSD induced developmental delay, hyperglycemic phenotypes and transcriptional disturbances related to insulin signaling. As the HSD intake from the egg stage was felt throughout the whole life cycle, which was more likely to go through the process of organ development and cell signaling pathways, sequentially affecting glucose levels and insulin sensitivity of adult flies. Furthermore, we raised HSD-fed flies with a concomitant or subsequent intervention of BLPs. All findings showed that BLPs ameliorated the symptoms of HSD-induced dysglycemia involving retardation of carbohydrate digestion and alleviation of insulin resistance. In brief, flies exposed to BLPs had weight loss, decreased body glucose and triglycerides levels, inhibited the activity of carbohydrate digestive enzymes, and restored over-production of insulin based on gene expression, compared with HSD-fed flies (see Figure 8). We hypothesized that BLPs were the potential to be developed as functional foods contributing to preventing and treating the abnormalities in T2DM. Further, the experiments using other animal models remain to be investigated, which helped deeply elucidate the hypoglycemic mechanism of BLPs.

## Data Availability

The original contributions presented in the study are included in the article/Supplementary Material, further inquiries can be directed to the corresponding authors.
