# Supplementary material for: Ameliorative effect of bayberry leaves proanthocyanidins on high sugar diet induced Drosophila melanogaster
Source: Front Pharmacol. 2022 Sep 15;13:1008580. doi: 10.3389/fphar.2022.1008580 (PMC9521571; doi:10.3389/fphar.2022.1008580)
Supplement: Supplementary file 1 [file DataSheet1.docx]

**Supplementary Materials**


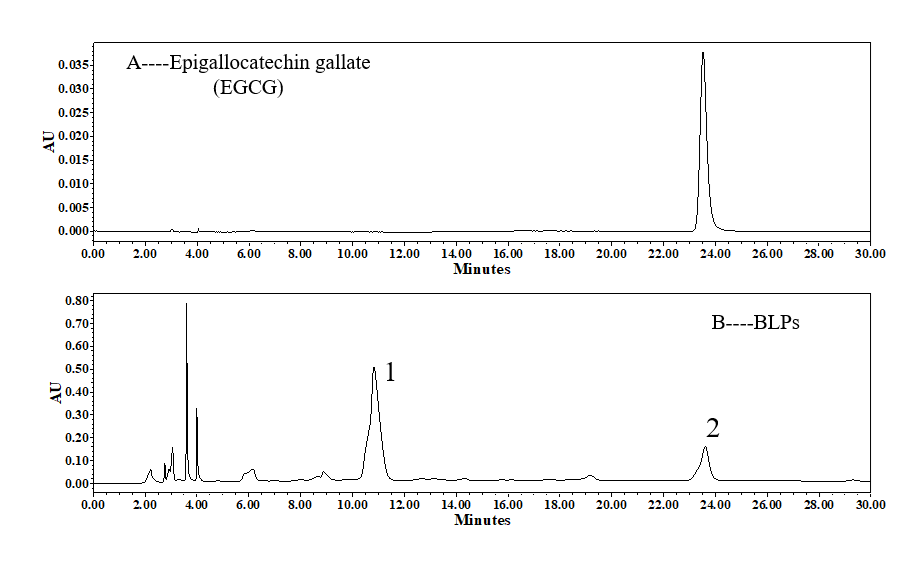


**Fig. S1.** HPLC chromatograms (detected at 280 nm) of standards of EGCG (A) and thiolyzed BLPs (B). Peak 1: EGCG cysteamine thioether (thio-EGCG); peak 2: EGCG.

**Table S2.** Fractions from BLPs and their identification from normal-phase preparative HPLC-ESI/MS and reverse-phase HPLC-ESI/MS.

| Fractions^a^ | Yield^b^ (mg/200mg) | MW (g/mol)^c^ | Tentative identification^d^ |
| --- | --- | --- | --- |
| 1 | 10.8±0.3 | 616 | myricetin deoxyhexoside-gallate |
| 2 | 4.6±0.1 | 744, 882 | (E)GC+(E)CG, 2(E)CG, 2(E)C+(E)GC |
| 3 | 8.6±0.3 | 762 | (E)GC+(E)GCG |
| 4 | 8.6±0.4 | 914 | 2(E)GCG |
| 5 | 4.3±0.2 | 1066 | 2(E)GC+(E)GCG |
| 6 | 8.7±0.2 | 1218 | (E)GC+2(E)GCG |
| 7 | 13.0±1.5 | 1371 | 3(E)GCG, 3(E)GC+(E)GCG |
| 8 | 19.6±0.6 | 1523 | 2(E)GC+2(E)GCG |
| 9 | 32.2±1.6 | 1675 | (E)GC+3(E)GCG |
| 10 | 43.8±1.0 | 1827 | 3(E)CG+(E)GCG, 4(E)GCG |

^a^ Fractions, Fractions and their identification from normal-phase preparative HPLC- ESI/MS and reverse-phase HPLC-ESI/MS.

^b^ Yield, the yield of one injection of preparative HPLC, that is, milligram per 200 milligrams BLPs.

^c^ MW, molecular weight.

^d^ Tentative identification, (E)GC, (E)GCG, (E)CG are abbreviations for (epi)gallocatechin, (epi)gallocatechin-3-O-gallate, (epi)catechin-3-O-gallate.

**
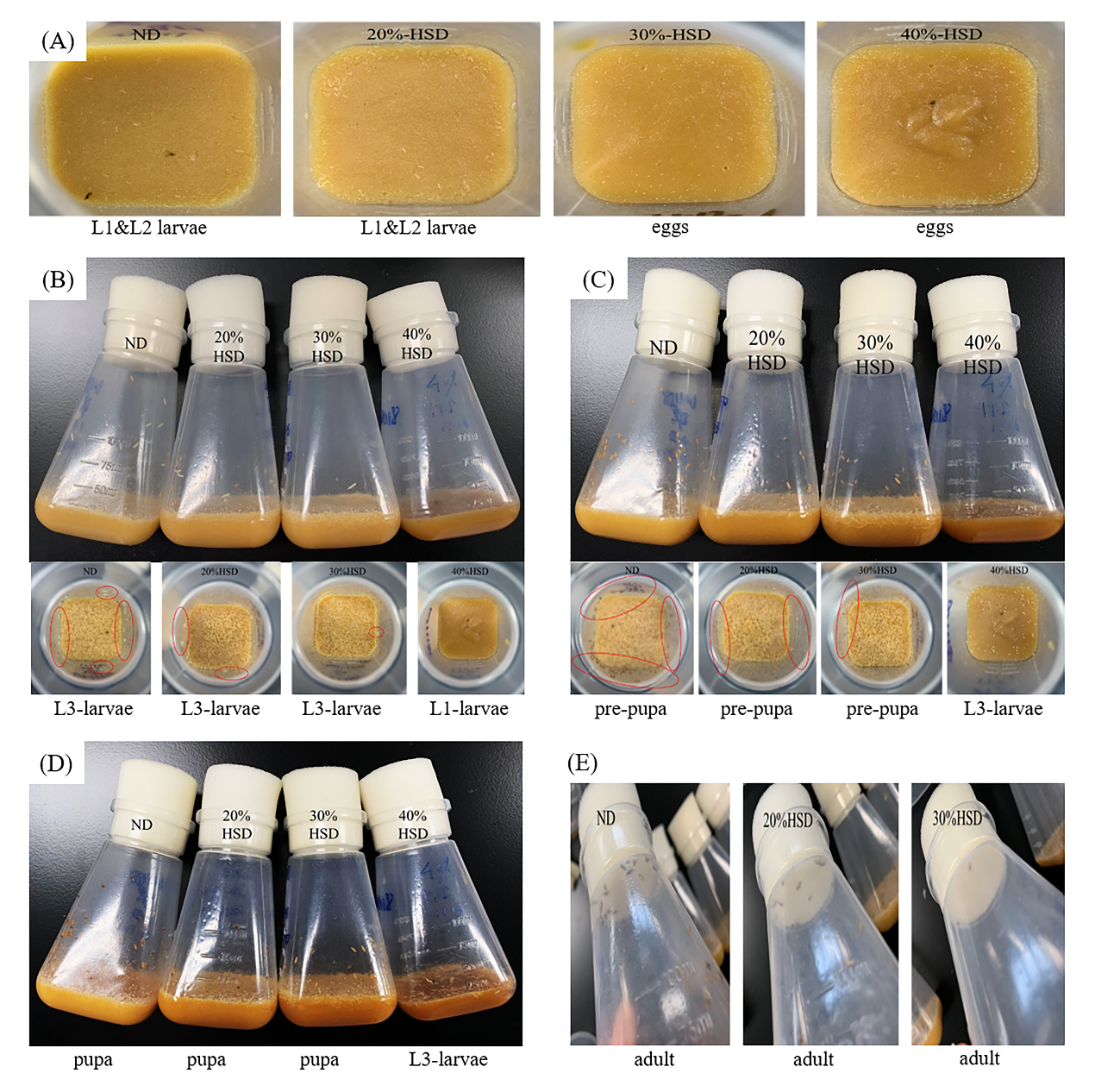
**

**Fig. S2.** Developmental rate of *Drosophila* from egg to adult lifecycle (egg-embryo-1^st^ instar larva-2^nd^ instar larvae-3^rd^ instar larvae-prepupa-pupa-adult) with normal diet (ND) and 20%-40% high-sugar diet (HSD) feeding. (A)-(E) represented the record on the 4th, 7th, 9th, 12th and 13th day after HSD treatment, corresponding to the first time observing L1&L2 larvae, L3 larvae, pre-pupa, pupa, and adult in the ND group. Meantime, developmental delay of 2-3 days in 30% HSD group. Here, 40% HSD feeding was observed stop-growing at the stage of L3 larvae and even cannot reach the pupal state and turn into flies in further thirty days.

**
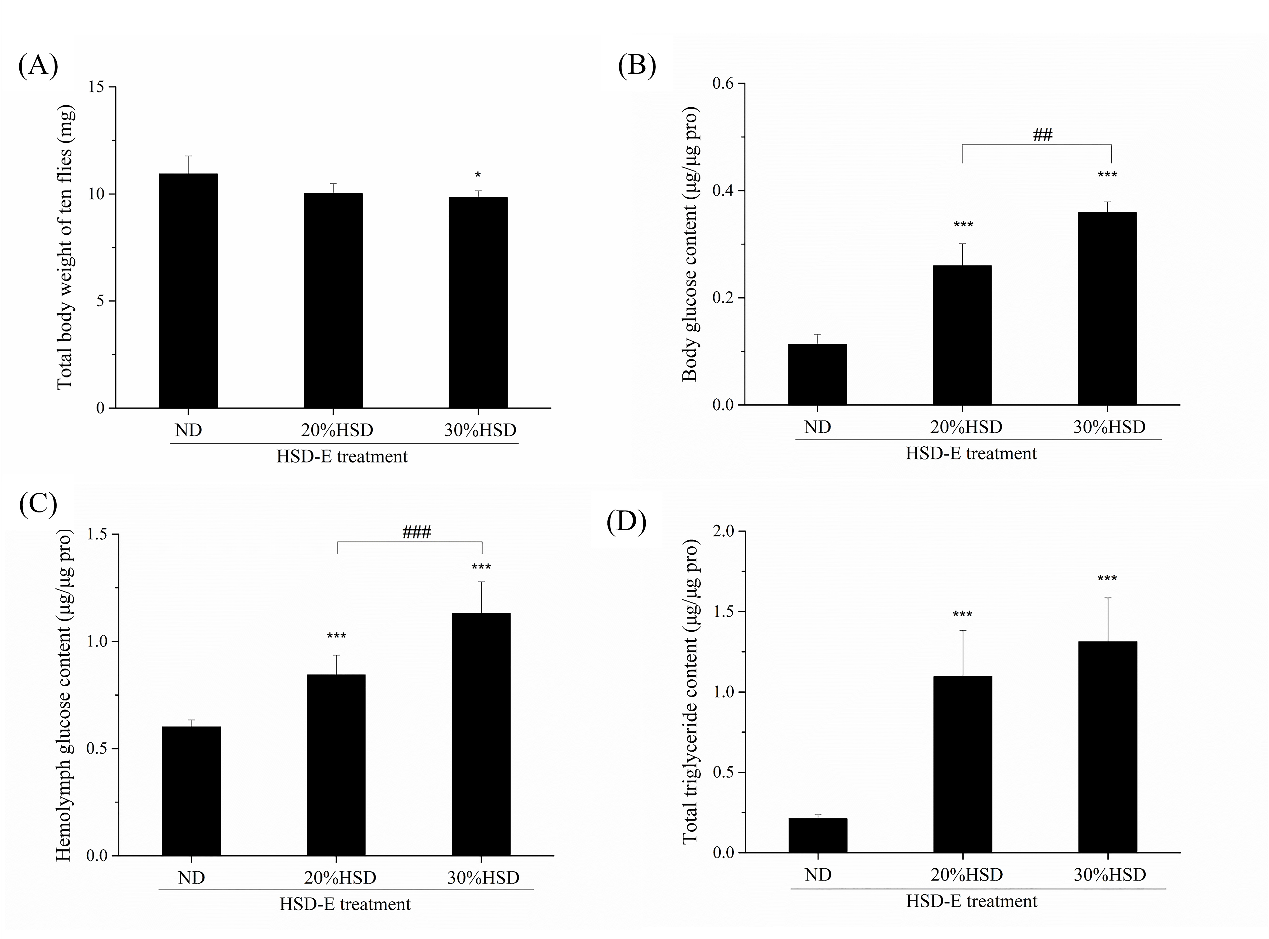
**

**Fig. S3.** Total weight (ten of flies) (A), body glucose content (B), hemolymph glucose content (C), and total triglyceride content (D) of flies fed on a high-sugar diet from egg-stage (HSD-E treatment). Significance is marked as * p < 0.05, ** p < 0.01 and ** p < 0.001 compared to the normal diet (ND) control, ## p < 0.01 and ### p < 0.001 between two HSD groups when represented with lines.

**
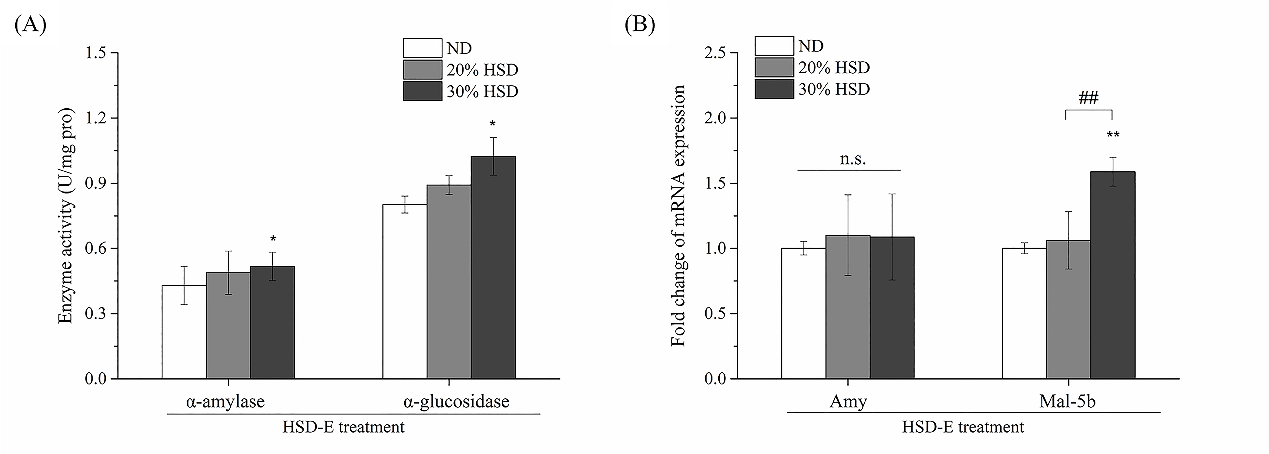
**

**Fig. S4.** α-Amylase and α-glucosidase activity (A) and mRNA expression change of *Amy* and *Mal-5b* genes (B) of flies fed on a high-sugar diet from egg-stage (HSD-E treatment). Significance is marked as * p < 0.05 and ** p < 0.01 compared to the ND control, ## p < 0.01 between two HSD groups when represented with lines, and n.s. represented no significance between groups here.





**Fig.S5.** mRNA expression of main genes related to glucose and lipid metabolism in flies fed on a high-sugar diet from egg-stage (HSD-E treatment). Significance is marked as * p < 0.05, ** p < 0.01 and ** p < 0.001 compared to the normal diet (ND) control group.

**Data Tables:**

**Table A.** Body weight (ten flies), body glucose content, and total triglyceride content of those flies fed on a diet containing BLPs at different concentrations in pre-treatment or post-treatment, respectively. (corresponding to the data in Fig.3)

| Treatment | Groups | Body weight of ten flies (mg) | Body glucose content (μg/μg pro) | Total triglyceride content (μg/μg pro) |
| --- | --- | --- | --- | --- |
| pre-treatment | ND | 10.63±0.35^a^ | 0.08±0.03^c^ | 0.15±0.04^c^ |
|  | HSD | 9.48±0.25^b^ | 0.32±0.02^a^ | 0.71±0.02^a^ |
|  | 0.1%BLPs/HSD | 9.36±0.42^bc^ | 0.20±0.04^b^ | 0.66±0.03^a^ |
|  | 0.2%BLPs/HSD | 9.03±0.71^bc^ | 0.19±0.03^b^ | 0.65±0.11^a^ |
|  | 0.5%BLPs/HSD | 8.82±0.60^c^ | 0.12±0.04^c^ | 0.37±0.10^b^ |
| post-treatment | ND+ND | 11.16±0.56^a^ | 0.61±0.06^b^ | 0.57±0.04^b^ |
|  | HSD+ND | 11.32±0.17^a^ | 0.71±0.03^a^ | 0.76±0.09^a^ |
|  | HSD+0.1%BLPs/ND | 11.06±0.48^a^ | 0.51±0.04^c^ | 0.45±0.07^c^ |
|  | HSD+0.2%BLPs/ND | 10.82±0.51^a^ | 0.40±0.04^d^ | 0.42±0.11^cd^ |
|  | HSD+0.5%BLPs/ND | 10.11±0.33^b^ | 0.38±0.03^d^ | 0.31±0.03^d^ |

Data (Mean ± SD) in the same column with different letters (a, b, c, d) were significantly different (*p* < 0.05).

**Table B.** α-Amylase activity and α-glucosidase activity of those flies fed on a diet containing BLPs at different concentrations in pre-treatment or post-treatment, respectively. (corresponding to the data in Fig.4)

| Treatment | Groups | α-Amylase activity (U/mg pro) | α-Glucosidase activity (U/mg pro) |
| --- | --- | --- | --- |
| pre-treatment | ND | 0.47±0.10^ab^ | 0.83±0.02^b^ |
|  | HSD | 0.50±0.05^ab^ | 1.16±0.10^a^ |
|  | 0.1%BLPs/HSD | 0.53±0.05^a^ | 0.35±0.01^c^ |
|  | 0.2%BLPs/HSD | 0.45±0.02^ab^ | 0.28±0.03^c^ |
|  | 0.5%BLPs/HSD | 0.40±0.04^b^ | 0.30±0.03^c^ |
| post-treatment | ND+ND | 0.73±0.06^b^ | 0.89±0.07^a^ |
|  | HSD+ND | 1.05±0.17^ab^ | 0.93±0.05^a^ |
|  | HSD+0.1%BLPs/ND | 1.17±0.37^a^ | 0.45±0.13^b^ |
|  | HSD+0.2%BLPs/ND | 0.95±0.12^ab^ | 0.45±0.04^b^ |
|  | HSD+0.5%BLPs/ND | 0.76±0.01^b^ | 0.58±0.03^b^ |

Data (Mean ± SD) in the same column with different letters (a, b, c, d) were significantly different (*p* < 0.05).

**Table C.** mRNA expression of *Amy* gene and *Mal-5b* gene of those flies fed on media containing BLPs at different concentrations in pre-treatment and post-treatment, respectively. (corresponding to the data in Fig.5)

| Treatment | Groups | Fold change of *Amy* mRNA expression | Fold change of *Mal* mRNA expression |
| --- | --- | --- | --- |
| pre-treatment | ND | 1.00±0.06^ab^ | 1.00±0.05^a^ |
|  | HSD | 1.05±0.08^ab^ | 1.03±0.04^a^ |
|  | 0.1%BLPs/HSD | 1.00±0.14^ab^ | 0.72±0.01^b^ |
|  | 0.2%BLPs/HSD | 1.12±0.03^a^ | 0.50±0.15^c^ |
|  | 0.5%BLPs/HSD | 0.94±0.09^b^ | 0.35±0.04^d^ |
| post-treatment | ND+ND | 1.01±0.18^a^ | 1.01±0.12^a^ |
|  | HSD+ND | 0.85±0.29^ab^ | 1.07±0.15^a^ |
|  | HSD+0.1%BLPs/ND | 0.66±0.03^bc^ | 0.57±0.04^b^ |
|  | HSD+0.2%BLPs/ND | 0.66±0.10^bc^ | 0.70±0.22^b^ |
|  | HSD+0.5%BLPs/ND | 0.47±0.01^c^ | 0.77±0.07^b^ |

Data (Mean ± SD) in the same column with different letters (a, b, c, d) were significantly different (*p* < 0.05).

**Table D.** mRNA expression of main genes associated with glucose metabolism of those flies fed on media containing BLPs at different concentrations. (corresponding to the data in Fig.6)

| Treatment | Groups | *dilp2* | *dilp3* | *InR* | *dAKT* | *dTOR* | *dFOXO* | *PEPCK* | *MAPK* |
| --- | --- | --- | --- | --- | --- | --- | --- | --- | --- |
| pre-treatment | ND | 1.00±0.02^bc^ | 1.04±0.33^b^ | 1.01±0.15^b^ | 1.01±0.16^b^ | 1.02±0.21^b^ | 1.00±0.11^a^ | 1.05±0.37^c^ | 1.00±0.07^b^ |
|  | HSD | 1.12±0.04^ab^ | 1.75±0.45^a^ | 1.38±0.10^a^ | 2.06±0.41^a^ | 1.56±0.23^a^ | 1.16±0.36^a^ | 3.06±0.33^a^ | 1.57±0.04^a^ |
|  | 0.1%BLPs/HSD | 1.21±0.13^a^ | 1.60±0.24^a^ | 0.85±0.10^b^ | 0.64±0.15^bc^ | 0.79±0.29^b^ | 0.35±0.20^b^ | 1.40±0.44^bc^ | 0.67±0.04^c^ |
|  | 0.2%BLPs/HSD | 0.83±0.16^c^ | 1.64±0.25^a^ | 0.52±0.10^c^ | 0.42±0.11^cd^ | 0.75±0.22^b^ | 0.28±0.07^b^ | 2.03±0.63^b^ | 0.42±0.07^d^ |
|  | 0.5%BLPs/HSD | 0.46±0.17^d^ | 1.59±0.16^a^ | 0.23±0.03^d^ | 0.12±0.00^d^ | 0.08±0.06^c^ | 0.10±0.07^b^ | 0.79±0.51^c^ | 0.15±0.06^e^ |
| post-treatment | ND+ND | 1.01±0.17^b^ | 1.01±0.15^b^ | 1.00±0.10^b^ | 1.00±0.06^a^ | 1.02±0.23^ab^ | 1.00±0.08^a^ | 1.00±0.06^b^ | 1.00±0.09^b^ |
|  | HSD+ND | 1.36±0.14^a^ | 1.88±0.67^a^ | 1.25±0.30^a^ | 1.05±0.08^a^ | 1.31±0.02^a^ | 0.80±0.15^b^ | 1.55±0.30^a^ | 1.72±0.33^a^ |
|  | HSD+0.1%BLPs/ND | 0.93±0.23^b^ | 1.46±0.07^ab^ | 0.17±0.04^c^ | 0.53±0.07^b^ | 1.11±0.24^ab^ | 0.18±0.01^d^ | 0.65±0.07^c^ | 1.47±0.17^a^ |
|  | HSD+0.2%BLPs/ND | 0.87±0.10^b^ | 1.22±0.19^b^ | 0.37±0.04^c^ | 0.53±0.06^b^ | 0.85±0.05^c^ | 0.49±0.05^c^ | 1.07±0.22^b^ | 1.10±0.04^b^ |
|  | HSD+0.5%BLPs/ND | 0.87±0.20^b^ | 1.15±0.30^b^ | 0.23±0.05^c^ | 0.41±0.05^c^ | 0.96±0.29^c^ | 0.54±0.03^c^ | 1.01±0.13^b^ | 0.71±0.08^c^ |

Data (Mean ± SD) in the same column with different letters (a, b, c, d) were significantly different (*p* < 0.05).

**Table E.** mRNA expression of main genes associated with lipid metabolism of those flies fed on media containing BLPs at different concentrations. (corresponding to the data in Fig.7)

| Treatment | Groups | *E78* | *SREBP* | *FAS* | *LSD* |
| --- | --- | --- | --- | --- | --- |
| pre-treatment | ND | 1.04±0.34^a^ | 1.03±0.28^a^ | 1.01±0.19^b^ | 1.00±0.10^b^ |
|  | HSD | 1.23±0.43^a^ | 1.20±0.30^a^ | 1.48±0.38^a^ | 2.09±0.38^a^ |
|  | 0.1%BLPs/HSD | 0.82±0.21^a^ | 0.48±0.16^b^ | 0.88±0.06^b^ | 1.30±0.22^b^ |
|  | 0.2%BLPs/HSD | 0.75±0.36^a^ | 0.89±0.33^ab^ | 1.37±0.09^a^ | 0.97±0.30^b^ |
|  | 0.5%BLPs/HSD | 0.10±0.06^b^ | 0.86±0.21^ab^ | 0.47±0.14^c^ | 0.06±0.03^c^ |
| post-treatment | ND+ND | 1.02±0.24^a^ | 1.01±0.12^b^ | 1.00±0.10^b^ | 1.01±0.17^b^ |
|  | HSD+ND | 1.08±0.38^a^ | 1.43±0.28^a^ | 1.75±0.09^a^ | 1.79±0.13^a^ |
|  | HSD+0.1%BLPs/ND | 0.51±0.32^bc^ | 0.73±0.15^b^ | 1.17±0.08^b^ | 0.49±0.07^c^ |
|  | HSD+0.2%BLPs/ND | 0.87±0.19^ab^ | 1.41±0.20^c^ | 1.18±0.11^b^ | 1.05±0.37^b^ |
|  | HSD+0.5%BLPs/ND | 0.33±0.07^c^ | 0.91±0.14^b^ | 1.02±0.22^b^ | 0.48±0.12^c^ |

Data (Mean ± SD) in the same column with different letters (a, b, c, d) were significantly different (*p* < 0.05).

**Table F.** Total weight (ten flies), body glucose content, hemolymph glucose content, and total triglyceride content of flies fed on a high-sugar diet from egg-stage (HSD-E treatment). (corresponding to the data in Fig.S3)

| Groups | Body weight of ten flies (mg) | Body glucose content (μg/μg pro) | Hemolymph glucose content (μg/μg pro) | Total triglyceride content (μg/μg pro) |
| --- | --- | --- | --- | --- |
| ND | 10.94±0.84 | 0.11±0.02 | 0.60±0.03 | 0.21±0.02 |
| 20%HSD | 10.02±0.46 | 0.26±0.04*** | 0.84±0.09*** | 1.10±0.29*** |
| 30%HSD | 9.83±0.31* | 0.36±0.02*** | 1.13±0.15*** | 1.31±0.27*** |

Significance is marked as * p < 0.05, ** p < 0.01 and ** p < 0.001 compared to the normal diet (ND) control.

**Table G.** α-Amylase and α-glucosidase activity and the mRNA expression change of *Amy* and *Mal-5b* genes of flies fed on a high-sugar diet from egg-stage (HSD-E treatment). (corresponding to the data in Fig.S4)

| Groups | α-Amylase activity (U/mg pro) | α-Glucosidase activity (U/mg pro) | Fold change of *Amy* mRNA expression | Fold change of *Mal* mRNA expression |
| --- | --- | --- | --- | --- |
| ND | 0.43±0.09 | 0.82±0.04 | 1.00±0.05 | 1.00±0.04 |
| 20%HSD | 0.49±0.10 | 0.89±0.04 | 1.10±0.31 | 1.06±0.22 |
| 30%HSD | 0.52±0.07* | 1.02±0.09* | 1.09±0.33 | 1.59±0.11** |

Significance is marked as * p < 0.05 and ** p < 0.01 compared to the ND control, and ## p < 0.01 between two HSD groups.

**Table H.** mRNA expression of main genes related to glucose and lipid metabolism in flies fed on a high-sugar diet from egg-stage (HSD-E treatment). (corresponding to the data in Fig.S5)

| Groups | *dilp2* | *dilp3* | *InR* | *PEPCK* | *dAKT* | *ACC* | *dTOR* | *dFOXO* | *FAS* | *LSD* | *SREBP* | *E78* |
| --- | --- | --- | --- | --- | --- | --- | --- | --- | --- | --- | --- | --- |
| ND | 1.02±0.20 | 1.01±0.19 | 1.00±0.10 | 1.01±0.13 | 1.01±0.18 | 1.01±0.13 | 1.02±0.24 | 1.00±0.08 | 1.02±0.25 | 1.01±0.15 | 1.01±0.15 | 1.01±0.18 |
| 20%HSD | 1.42±0.21* | 3.92±1.33* | 1.05±0.15 | 2.12±0.55 | 1.41±0.18** | 0.70±0.24 | 0.98±0.24 | 0.84±0.17 | 1.33±0.16 | 1.07±0.30 | 1.37±0.30 | 1.03±0.25 |
| 30%HSD | 1.93±0.28** | 4.35±0.36*** | 3.31±0.07** | 3.66±0.49** | 1.95±0.46** | 0.06±0.04*** | 1.61±0.10* | 0.75±0.03 | 1.47±0.13* | 2.87±0.96* | 1.52±0.18* | 1.59±0.05* |

Significance is marked as * p < 0.05, ** p < 0.01 and ** p < 0.001 compared to the normal-diet (ND) control group.
